# Control of the expiratory flow in a lung model and in healthy volunteers with an adjustable flow regulator: a combined bench and randomized crossover study

**DOI:** 10.1186/s12931-021-01886-7

**Published:** 2021-11-14

**Authors:** Johannes Schmidt, Anna Martin, Christin Wenzel, Jonas Weber, Steffen Wirth, Stefan Schumann

**Affiliations:** 1 Department of Anesthesiology and Critical Care, Medical Center - University of Freiburg, Faculty of Medicine, University of Freiburg, Hugstetter Str. 55, 79106 Freiburg, Germany; 2 Department of Anesthesia, Intensive Care and Pain Medicine, Medical Center of the German Accident Insurance Institution, Murnau, Germany

**Keywords:** Expiratory flow regulation, Expiratory resistive load, Pursed-lips breathing, Spontaneous breathing, Mandatory ventilation

## Abstract

**Background:**

Pursed-lips breathing (PLB) is a technique to attenuate small airway collapse by regulating the expiratory flow. During mandatory ventilation, flow-controlled expiration (FLEX), which mimics the expiratory flow course of PLB utilizing a digital system for measurement and control, was shown to exert lung protective effects. However, PLB requires a patient’s participation and coordinated muscular effort and FLEX requires a complex technical setup. Here, we present an adjustable flow regulator to mimic PLB and FLEX, respectively, without the need of a patient’s participation, or a complex technical device.

**Methods:**

Our study consisted of two parts: First, in a lung model which was ventilated with standard settings (tidal volume 500 ml, respiratory rate 12 min^−1^, positive end-expiratory pressure (PEEP) 5 cmH_2_O), the possible reduction of the maximal expiratory flow by utilizing the flow regulator was assessed. Second, with spontaneously breathing healthy volunteers, the short-term effects of medium and strong expiratory flow reduction on airway pressure, the change of end-expiratory lung volume (EELV), and breathing discomfort was investigated.

**Results:**

In the lung model experiments, expiratory flow could be reduced from − 899 ± 9 ml·s^−1^ down to − 328 ± 25 ml·s^−1^. Thereby, inspiratory variables and PEEP were unaffected. In the volunteers, the maximal expiratory flow of − 574 ± 131 ml·s^−1^ under baseline conditions was reduced to − 395 ± 71 ml·s^−1^ for medium flow regulation and to − 266 ± 58 ml·s^−1^ for strong flow regulation, respectively (p < 0.001). Accordingly, mean airway pressure increased from 0.6 ± 0.1 cmH_2_O to 2.9 ± 0.4 cmH_2_O with medium flow regulation and to 5.4 ± 2.4 cmH_2_O with strong flow regulation, respectively (p < 0.001). The EELV increased from baseline by 31 ± 458 ml for medium flow regulation and 320 ± 681 ml for strong flow regulation (p = 0.033). The participants rated breathing with the flow regulator as moderately uncomfortable, but none rated breathing with the flow regulator as intolerable.

**Conclusions:**

The flow regulator represents an adjustable device for application of a self-regulated expiratory resistive load, representing an alternative for PLB and FLEX. Future applications in spontaneously breathing patients and patients with mandatory ventilation alike may reveal potential benefits.

*Trial registration*: DRKS00015296, registered on 20th August, 2018; URL: https://www.drks.de/drks_web/setLocale_EN.do.

**Supplementary Information:**

The online version contains supplementary material available at 10.1186/s12931-021-01886-7.

## Background

Control of the expiration by pursed-lips breathing (PLB) is a well-known technique to attenuate small airway collapse [1]. In patients with obstructive pulmonary disease exhalation against an artificial airway resistance like PLB increased functional residual capacity [2], reduced hyperinflation [3], and improved airway clearance [4]. Further, the breathing pattern during PLB is associated with a decreased respiratory rate (RR) [5, 6]. Comparable effects were shown for breathing with an externally applied positive expiratory pressure [7]. After thoracic and abdominal surgery, chest physiotherapy with positive expiratory pressure improved lung function and reduced post-operative pulmonary complications [8].

A different approach to control the expiration is the so-called flow-controlled expiration (FLEX), which creates an almost constant expiratory flow with an external, variable resistor which is operated by a digital system for measurement and control of the tidal flow [9]. FLEX was shown to exert lung protective effects in an animal model of acute respiratory distress syndrome during mandatory ventilation [10], which may be attributed to a recruiting effect of lung tissue in dorsal lung regions [11]. Lung healthy volunteers reported a lower breathing discomfort with FLEX than with external application of positive expiratory pressure [12]. Additionally, FLEX did not constrain physical performance of healthy volunteers during exercise [13]. For mandatory ventilation during general anaesthesia, FLEX could shift the ventilation from ventral to dorsal lung regions in lung healthy patients undergoing cranial surgery [14].

The above-mentioned techniques imply several drawbacks. PLB depends on patient’s participation with strength and coordination. Moreover, PLB and its external equivalents cannot be applied during mandatory ventilation when the biological upper airways are bridged by the artificial airways. While FLEX may handle these points, it requires a complex technical setup which restrains a wider use. Further, during application in volunteers, the effects of FLEX could be partially compensated by deliberately increasing the maximal expiratory flow [12].

Here, we present an adjustable flow regulator to mimic PLB and FLEX, respectively, without the need for a patient’s effort, consciousness, or a complex technical setup. We hypothesized, that the expiratory flow could be reduced depending on the adjustments of the flow regulator during mandatory ventilation and during spontaneous breathing. Therefore, we investigated the capabilities of the flow regulator in a mechanically ventilated lung model and tested its application with moderate and strong expiratory flow limitation in healthy volunteers.

## Methods

**Fig. 1:**
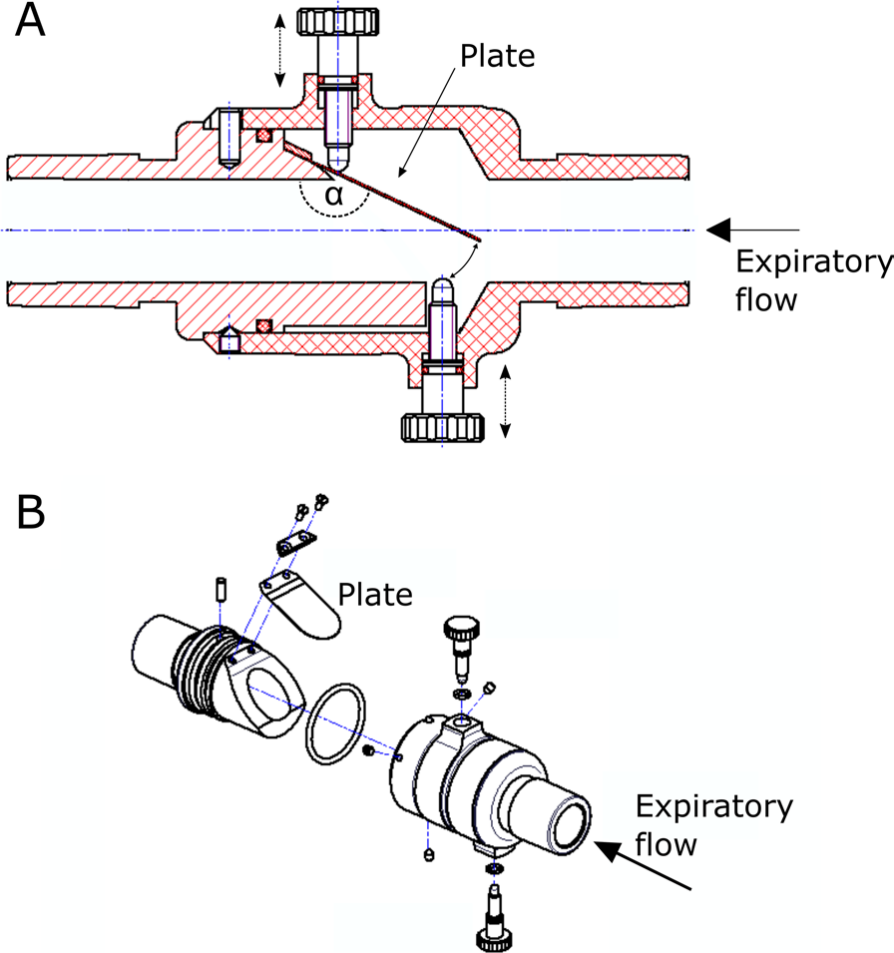
Cross section (**A**) and 3-dimensional construction (**B**) of the flow regulator. Note the diagonally suspended plate with its suspension angle α, which can be adjusted by the screw above the plate. The maximal deflection of the plate is adjusted by the screw below the plate

All experiments were conducted in the Department of Anesthesiology and Critical Care of the Medical Center of the University of Freiburg. The flow regulator consists of a tubular conductor containing a flexible plate, which is placed diagonally at an adjustable angle α (Fig. 1). The plate is made of fibre glass and coated with silicone. At its suspension, it is 0.11 mm thick and possesses mechanical characteristics of a spring: a certain gas flow bends it downwards until it partially occludes the expiratory airway. A small remaining cross-sectional area is retained to allow a residual flow even if the plate is maximally deflected. The higher the deflection of the plate, the higher is the artificial airway resistance. With decreasing gas flow, the plate turns back towards its starting position, and flow resistance is lowered. There are two mechanisms which allow to adjust the behaviour of the flow regulator: First, the angle of the plate is set by a screw placed at the plate’s suspension. With steeper angle the flow threshold which triggers a plate response decreases. Second, the maximal partial airway occlusion can be set by adjusting the plate stop and thus the remaining cross-sectional area at maximal closure. The mechanical behaviour of the flow regulator and the described adjustments were investigated a priori and the respective data are available as Additional file 1.

**Fig. 2:**
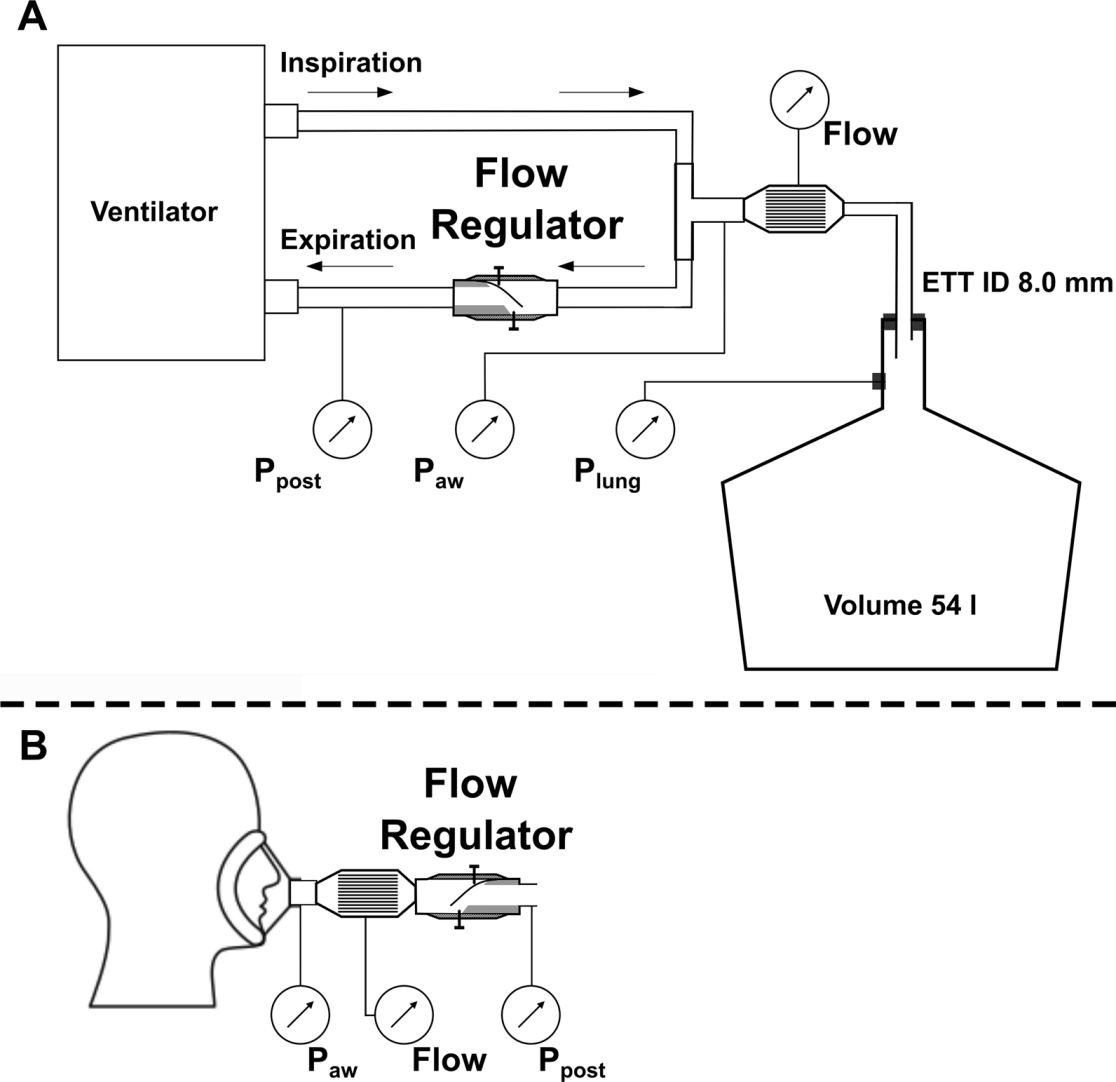
Schematic drawing of the setup for the measurements with the lung model (**A**) and with healthy volunteers (**B**). *ETT* endotracheal tube; *ID* inner diameter; *P*_*lung*_ pressure inside the lung model, *P*_*aw*_ airway pressure at the Y-piece; *P*_*post*_ pressure behind the flow regulator

Our study was divided into two parts. First, we investigated if the flow regulator can be used to reduce the maximal expiratory flow in a mechanically ventilated lung model (Fig. 2A) without increasing the end-expiratory pressure inside the lung model above the set positive end-expiratory pressure (PEEP). Therefore, the neck of a glass bottle providing a compliance of 54 ml·cmH_2_O^−1^ was intubated with a standard endotracheal tube with an inner diameter of 8 mm (Medtronic GmbH, Meerbusch, Germany). The endotracheal tube was connected to a standard ICU ventilator (Evita V500, Dräger medical, Lübeck, Germany) via a breathing circuit with the flow regulator integrated in the expiratory limb. The volume-controlled ventilation was applied with tidal volume (V_T_) 500 ml, respiratory rate (RR) 12 min^−1^, inspiratory time (T_In_) 1.8 s, expiratory time (T_Ex_) 3.2 s, and PEEP 5 cmH_2_O. Flow regulation was adjusted by reducing the aperture at maximal closure from 3.15 mm down to 0.35 mm in 17 steps. For each setting, flow and pressure were measured for 12 breaths. Pressure and flow signals were used to calculate the maximal expiratory flow, the mean lung pressure (P_lung_), and the mean expiratory flow resistance. To detect potential auto-PEEP, end-expiratory pressure in the lung was calculated as the P_lung_ during the last 50 ms of the expiratory phase. For each setting, measurements were repeated three times after complete dismantling and reassembly of the setup.

In the second part of our study, we investigated the effects of the reduced maximal expiratory flow on the respiration parameters and on the perceived breathing discomfort in healthy volunteers. Ethical approval for this study was obtained from the Ethics Committee of the University of Freiburg (file reference EK-319/17). The study was registered in the German Register for Clinical Trials (File reference: DRKS00015296) before inclusion of the first test person. Informed consent was obtained from the volunteers before participation in the study. Exclusion criteria were age < 18 years, pregnancy, known history of pulmonary or cardiac diseases. Additional to airway pressure and flow, electrical impedance tomography (EIT) was recorded with a 16-electrode belt placed at mid-thoracic level with a sampling rate of 50 Hz (PulmoVista 500, Dräger medical, Lübeck, Germany). Subjects were placed in a sitting position and wearing a tight-fitting face mask. For accommodation, they breathed for several minutes via the mask without the flow regulator connected. The last 3 min of this accommodation were recorded as a baseline measurement. Then the flow regulator was connected (Fig. 2B) and two measurements of 3 min, one with medium and one with high flow regulation, in randomized order (determined by coin flip) were recorded: Expiratory flow regulations were defined as a reduction the of baseline maximal expiratory flow by 25% (‘Medium’), respectively by 50% (‘Strong’). After each measurement, the subjects rated breathing discomfort on a five-point categorical scale as comfortable, slightly uncomfortable, moderately uncomfortable, very uncomfortable, or intolerable. Interruption of the measurement by the subject was rated as intolerable as well. Further endpoints were the maximal expiratory flow, T_In_, T_Ex_, RR, minute volume (MV), V_T_, and mean expiratory airway resistance. The thoracic electrical impedance amplitude of the baseline measurement was calibrated with the calculated expiratory volume. Then, the change of the end-expiratory lung volume (∆EELV) from baseline measurement to both expiratory flow control settings was determined as described previously [15, 16].

For both parts of the study, flow was determined utilizing a pneumotachograph (Type Fleisch 1, Dr. Fenyves und Gut, Hechingen, Germany). Airway pressures were measured in front of and behind the flow regulator. For the part which utilized a lung model, an additional pressure measurement inside the lung model was established. The flow and pressure signals were recorded with a rate of 200 Hz by dedicated software (Labview Version 7.01, Austin, Texas, USA). Data analyses were done offline utilizing MATLAB (Version 2018a, The MathWorks Inc., Natick, MA, USA).

### Statistics

For the volunteer study, an a-priori sample size estimation, based on the assumed proportion of participants tolerating the set flow limitation of 0.9 and a total width of the confidence interval of 0.25, resulted in 30 participants needed to compare group proportions at a confidence level of 95%.

Continuous data were assessed for a Gaussian distribution with the Lilliefors test. For the statistical analyses of the volunteer study, a linear mixed model with the setting (‘Baseline’, ‘Medium’, ‘Strong’) as fixed effects and the subject as well as the randomization sequence as random intercepts was fitted to the data. A second model without a fixed effect was fitted and both models were compared by theoretical likelihood ratio to test for a significant difference. Post-hoc testing was omitted in favour of the calculation of the 95% confidence interval for the effect size (mean difference). Categorical data were assessed by a Chi square test. A p-value < 0.05 was considered significant. Data are presented as mean and standard deviation if not declared otherwise.

## Results

**Fig. 3:**
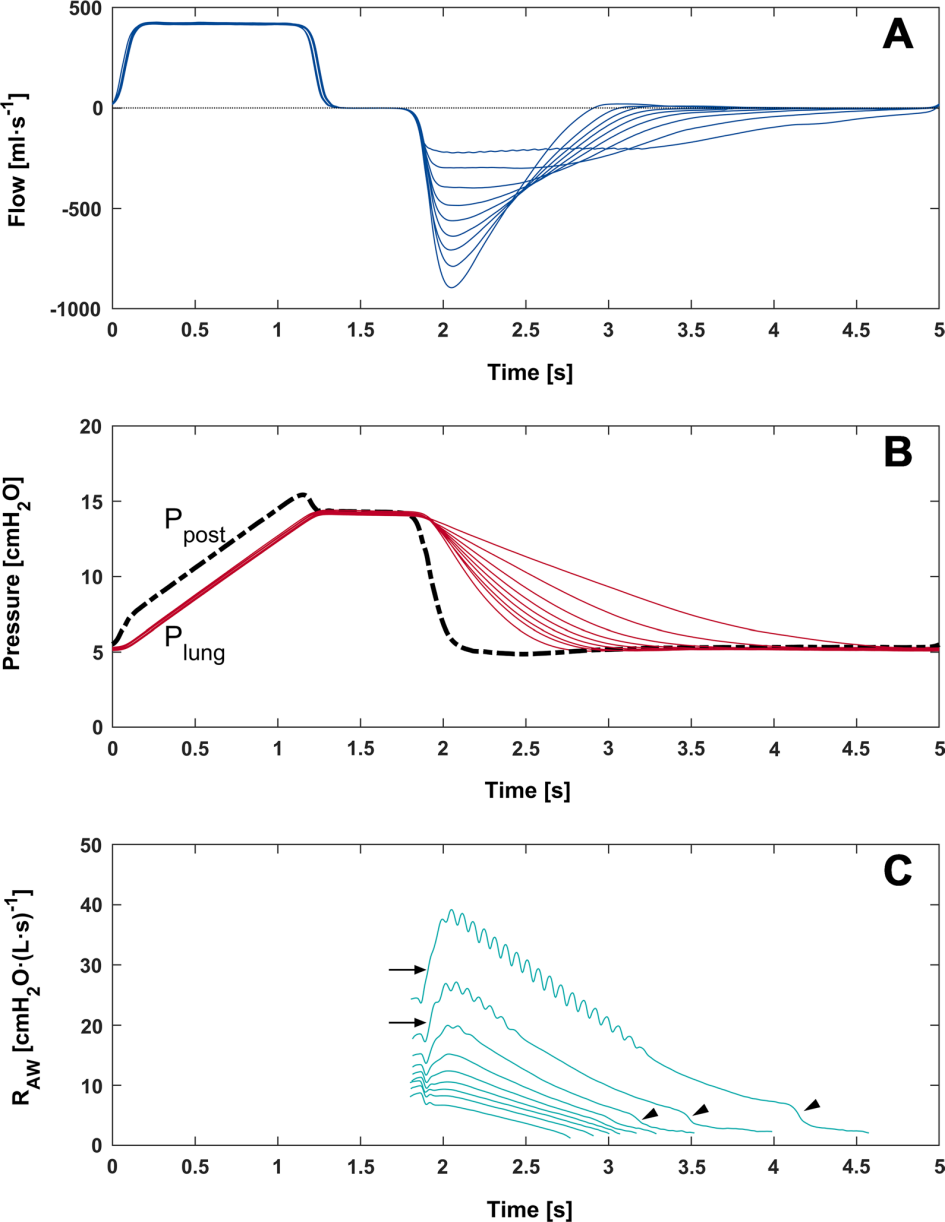
**A** Airway flow, **B** lung pressure (P_lung_), and **C** expiratory airway resistance (R_AW_) for an exemplary set of 9 different settings with decreasing aperture of the flow regulator during mandatory ventilation of a lung model. For each setting, one ventilation cycle is plotted beginning at the axis origin. Note that the inspiratory phase is unchanged by the different settings. In **B**, the pressure behind the flow regulator (P_post_) is depicted as dashed line. In **C**, abrupt rise in R_AW_ (arrows) indicates the plate deflection, abrupt drop in R_AW_ (arrow heads) indicates the backswing of the plate

In the mechanically ventilated lung model, maximal expiratory flow could be regulated from − 899 ± 9 ml·s^−1^ without regulation down to − 328 ± 25 ml·s^−1^ (Table 1). Maximal inspiratory pressure and end-expiratory pressure measured inside the lung model were unaffected from the expiratory flow regulation. Mean P_lung_ increased from 8.1 ± 0.01 without to maximally 8.8 ± 0.08 cmH_2_O with flow regulation (Fig. 3).

**Table 1:** Respiratory variables for ventilation of the lung model

Aperture setting [mm]	EFmax [ml·s^−1^]	R_total_ [cmH_2_O·L^−1^·s^−1^]	R_FR_ [cmH_2_O·L^−1^·s^−1^]	EEP_L_ [cmH_2_O]	P_Lmean_ [cmH_2_O]
BL	− 899 ± 9	5 ± 0.1	0.12 ± 0.00	5.28 ± 0.02	8.07 ± 0.01
3.150	− 809 ± 7	6 ± 0.05	1.27 ± 0.02	5.27 ± 0.03	8.12 ± 0.03
2.975	− 800 ± 8	6 ± 0.04	1.33 ± 0.02	5.27 ± 0.02	8.13 ± 0.02
2.800	− 794 ± 10	6 ± 0.07	1.41 ± 0.01	5.27 ± 0.02	8.13 ± 0.02
2.625	− 789 ± 8	6 ± 0.04	1.46 ± 0.00	5.27 ± 0.02	8.13 ± 0.02
2.450	− 780 ± 8	6 ± 0.03	1.60 ± 0.00	5.27 ± 0.02	8.14 ± 0.03
2.275	− 770 ± 9	6 ± 0.04	1.71 ± 0.01	5.27 ± 0.02	8.15 ± 0.02
2.100	− 755 ± 9	6 ± 0.07	1.88 ± 0.01	5.26 ± 0.04	8.15 ± 0.03
1.925	− 742 ± 7	6 ± 0.03	1.99 ± 0.02	5.25 ± 0.02	8.13 ± 0.03
1.750	− 724 ± 6	7 ± 0.05	2.18 ± 0.02	5.23 ± 0.03	8.12 ± 0.03
1.575	− 701 ± 4	7 ± 0.02	2.43 ± 0.03	5.22 ± 0.03	8.14 ± 0.03
1.400	− 672 ± 4	7 ± 0.05	2.76 ± 0.02	5.21 ± 0.02	8.15 ± 0.04
1.225	− 640 ± 2	7 ± 0.06	3.12 ± 0.06	5.20 ± 0.03	8.17 ± 0.01
1.050	− 604 ± 4	8 ± 0.05	3.55 ± 0.03	5.15 ± 0.02	8.17 ± 0.03
0.875	− 542 ± 4	8 ± 0.08	4.39 ± 0.11	5.11 ± 0.02	8.21 ± 0.02
0.700	− 452 ± 8	10 ± 0.12	5.88 ± 0.24	5.09 ± 0.02	8.32 ± 0.03
0.525	− 379 ± 8	11 ± 0.13	7.62 ± 0.18	5.06 ± 0.02	8.46 ± 0.05
0.350	− 328 ± 25	12 ± 0.19	10.68 ± 0.63	5.06 ± 0.02	8.78 ± 0.08

*Fmax* maximal expiratory flow; *R*_*total*_ total airway resistance; *R*_*FR*_ resistance of the flow regulator; *EEP*_*L*_ end-expiratory lung pressure; *P*_*Lmean*_ mean lung pressure

In total, 31 volunteers were included in the volunteer study. Data from one test person were excluded after the experiment due to incomplete data recordings. The demographic characteristics of the remaining participants are presented in Table 2.

**Table 2:** Demographic characteristics of the participating volunteers

Demographic characteristic	
Sex [m/f]	16/14
Age [years]	31 [18–64]
Height [cm]	176 [157–199]
Weight [kg]	71 [55–108]
BMI [kg·m^−2^]	23 [18–28]
Smoker/non-smoker	2/28

Data presented as n or mean [range]

*BMI* body mass index

**Fig. 4:**
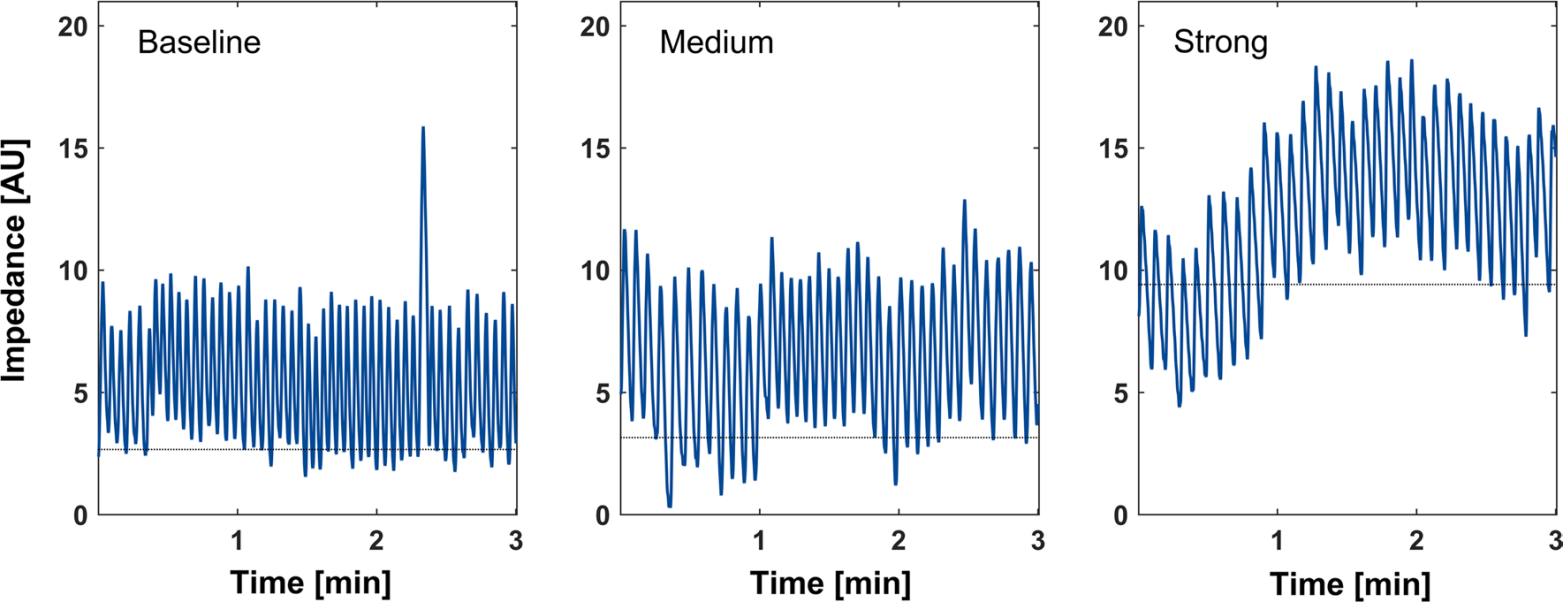
Thoracic electrical impedance variation expressed in arbitrary units (AU) of one volunteer during spontaneous breathing without flow regulation (Baseline, left), with medium flow regulation (center) and with strong flow regulation (right)

The respiratory variables are summarized in Table 3. Compared to baseline conditions, the maximal expiratory flow was reduced from − 574 ± 131 ml·s^−1^ to − 395 ± 71 ml·s^−1^ for medium flow regulation and to − 266 ± 58 ml·s^−1^ for strong flow regulation, respectively (p < 0.001). Accordingly, expiratory airway resistance increased from 1.9 ± 0.2 cmH_2_O·L^−1^·s^−1^ to 8.5 ± 3.1 cmH_2_O·L^−1^·s^−1^ for medium flow regulation and to 23.1 ± 9.3 cmH_2_O·L^−1^·s^−1^ for strong flow regulation, respectively (p < 0.001). Mean airway pressure increased from 0.6 ± 0.1 cmH_2_O to 2.9 ± 0.4 cmH_2_O for medium flow regulation and to 5.4 ± 2.4 cmH_2_O for strong flow regulation, respectively (p < 0.001). EELV increased by 31 ± 458 ml for medium flow regulation and by 320 ± 681 ml for high flow regulation compared to baseline (p = 0.033, Fig. 4).

**Table 3:** Breathing characteristics of the volunteers

	Baseline (Mean ± STD)	Medium flow regulation (Mean ± STD)	High flow regulation (Mean ± STD)	Effect size medium flow regulation (MD, 95% CI)		Effect size high flow regulation (MD, 95% CI)		p
EFmax [ml·s^−1^]	− 574 ± 131	− 395 ± 71	− 266 ± 58	180	[154, 205]	309	[283, 334]	< 0.001
R_AW_ [cmH_2_O·L^−1^·s^−1^]	1.9 ± 0.2	8.5 ± 3.1	23.1 ± 9.3	6.6	[4, 9.1]	21.2	[18.6, 23.7]	< 0.001
P_AW_ mean [cmH_2_O]	0.6 ± 0.1	2.9 ± 1.4	5.4 ± 2.4	2.2	[1.6, 2.9]	4.7	[4.1, 5.4]	< 0.001
P_AW_ max [cmH_2_O]	1.1 ± 0.3	4.8 ± 2.4	7.7 ± 2.9	3.8	[2.9, 4.6]	6.7	[5.8, 7.5]	< 0.001
V_T_ [ml]	1050 ± 360	1100 ± 420	1080 ± 460	60	[− 40, 150]	40	[− 60, 130]	0.47
MV [L·min^−1^]	11.6 ± 2.1	10.8 ± 2.1	9.1 ± 2.2	− 0.9	[− 0.3, − 1.4]	− 2.6	[− 2, − 3.1]	0.004
RR [min^−1^]	12 ± 4	11 ± 4	10 ± 4	− 1	[− 2, 0]	− 3	[− 3, − 2]	0.011
T_In_ [s]	2.3 ± 1.0	2.3 ± 1.1	2.1 ± 1.0	0	[− 0.1, 0.2]	− 0.3	[− 0.4, − 0.1]	0.053
T_Ex_ [s]	3 ± 0.9	3.7 ± 1.3	5 ± 2.1	0.7	[0.3, 1.1]	2	[1.5, 2.4]	0.004
∆EELV [ml]	n.a	31 ± 458	320 ± 681	31	[− 159, 222]	320	[130, 511]	0.033

*MD* mean difference; *CI* confidence interval; *EFmax* maximal expiratory flow; *R*_*AW*_ airway resistance; *P*_*AW*_ airway pressure; *V*_*T*_ tidal volume; *MV* minute volume; *RR* respiratory rate; *T*_*In*_ inspiratory time; *T*_*Ex*_ expiratory time; ∆*EELV* change of the end-expiratory lung volume

All participants finished the experiment without interruption and none of the participants rated the flow regulation as intolerable. With medium and strong flow regulation, the breathing discomfort was rated higher compared to baseline (Table 4; ‘medium’ vs. baseline p = 0.010 and ‘strong’ vs. baseline p < 0.001, respectively) and strong flow regulation was rated as more uncomfortable as medium flow regulation (p = 0.002).

**Table 4:** Rating of the breathing comfort by the volunteers

Setting	Comfortable [n]	Slightly uncomfortable [n]	Moderately uncomfortable [n]	Very uncomfortable [n]	Intolerable n]
Baseline	12	16	2	0	0
Medium flow regulation	11	12	16	1	0
Strong flow regulation	1	10	10	9	0

Chi square test: baseline vs. medium p = 0.010; baseline vs. high p < 0.001; medium vs. high p = 0.002

## Discussion

The main findings of this study can be summarized as follows: with mandatory ventilation an almost constant expiratory flow was achievable in a physical model. Thereby, incomplete expiration with the risk of dynamic hyperinflation was not observed. In healthy volunteers, reduction of the maximal expiratory flow increased EELV but did not cause an intolerable breathing discomfort.

To our knowledge this is the first study to demonstrate an increase of the EELV in volunteers spontaneously breathing against an externally applied airway resistance device. Other changes in the breathing pattern with flow regulation were comparable to previous studies.

The novelty of the presented flow regulator consists of three categories. First, in contrast to already available resistors the resulting airway resistance is regulated by the slowing expiratory flow. When expiratory flow is high, the flow regulator’s airway occlusion plate is deflected and provides a high resistance. As soon as the flow rate falls below a certain limit, the plate swings back resulting in a drop of resistance and thus the residual volume can be expired at minimal resistance (Fig. 3). Second, the adjustability concerning flow trigger and aperture allow for a wide applicability without the need to change devices or parts of the flow regulator. By the wide range of adjustments, applicability for spontaneous breathing as well as for mandatory ventilation was possible. Thereby, the device’s behaviour may be adjusted during the use. Third, the easy to use and intuitive handling may facilitate usage by health care professionals and patients as well.

Regulation of the expiratory flow via PLB or via application of an external resistive load is widely accepted as part of pulmonary rehabilitation programs for patients with pulmonary diseases [17, 18]. It may attenuate pulmonary hyperinflation [19], small airway collapse [20–22], and may relief symptoms of dyspnea [23, 24], which makes it a valuable emergency tool exerted directly by the patient [25, 26]. However, some patients with obstructive lung diseases do not adopt PLB and do not experience a relief of dyspnea. Especially under stress, which may be caused by the dyspnea itself, the performance of PLB may be unsuccessful because PLB usually depends on a strength and coordination effort which may be small but anyway overtaxing the patient in this specific situation [27]. Additionally, the performance of PLB is limited to several breathing cycles [27]. Combined with a mouthpiece or a tight-fitting facemask, the here presented flow regulator may have advantages over PLB and other devices for creating a resistive load, because the patient may adjust the settings during the use. Our study showed that the breathing discomfort increased with higher flow regulation, but interruption of the intervention or rating as intolerable was never observed. One might speculate that ongoing training with the device and adaptation to its effect would attenuate the perceived discomfort and moreover, the current requirement of expiratory resistance in a situation of exacerbation may increase the tolerability.

Mandatory ventilation of patients suffering from COPD can be challenging. General anaesthesia and muscle paralysis will promote the formation of atelectasis on the one hand and will enhance air trapping on the other hand. Taken together, the inhomogeneity of the lung tissue will increase, and one might speculate, that mimicking PLB may be beneficial. In fact, the rationale to regulate the expiratory flow is based on a multitude of observations. Previously shown beneficial effects of regulated expiratory flow include improved gas exchange as well as higher fraction of aerated lung tissue in healthy lungs and in ARDS lungs in an animal model [28, 29], higher EELV while ventilating with the same PEEP [16], a more homogeneous ventilation distribution in a multi-compartmental lung model [30], and reduced lung tissue damage after ventilation of ARDS lungs [10, 11]. However, the technical solutions that offer control of the expiratory flow are so far limited to experimental use, since a widespread availability of such devices does currently not exist. Adding a simple resistor to the expiratory limb of the ventilator increases the time constant of the expiratory flow curve, and thus lengthens the expiratory flow time without modifying the exponential character of lung emptying. However, previous studies indicate that for inhomogeneous lung compartments which share a high common expiratory flow resistance, a homogenization of the expiratory pressure distribution and hence a reduction of shear stress between the compartments is possible [30]. This almost constant expiratory flow could be observed in the present study. Nevertheless, a simple resistor does not offer the opportunity to individualize the flow regulation, and would furthermore per se lengthen the required expiration time by increasing the expiratory time constant. In contrast, the here presented device adds the feature that the expiratory airway resistance decreases with slowing gas flow, thus facilitated the end expiratory gas flow which counteracts the risk of creating auto-PEEP. Consistently, auto-PEEP could not be detected in this study.

## Limitations

The control range for the flow trigger is limited to a minimum initial expiratory flow of approximately 300 ml·s^−1^ (Additional File 1). A previous study showed that the initial expiratory flow in healthy subjects at rest is − 426 ± 110 ml·s^−1^ [12]. Based on this observation and regarding the nature of a Gaussian distribution, in ~ 15% of healthy subjects (flow lower than − 426 ml·s^−1^ − 110 ml·s^−1^ = 316 ml·s^−1^) it may be impossible to trigger a plate deflection with the current design of the flow regulator. In such cases, a different geometrical design would be needed. Additionally, there are other factors which may influence the mechanical characteristics of the plate, such as temperature, gas composition (as humidity, volatile anesthetics, nitric oxide), gravity, and mechanical degradation. The plate’s thickness at its suspension influences its deflection behavior and backswing as well. The examination of these factors was not part of the study.

During mandatory ventilation of the physical model, auto-PEEP did not occur in our study. However, the application of any resistive device in individual patients may create auto-PEEP and special attention is needed to avoid dynamic hyperinflation. In a clinical setting, where lung pressure cannot be measured directly, auto-PEEP can be detected by persisting end-expiratory flow. Application of the flow regulator distributes expiratory flow over the duration of expiration and could thus potentially prevent from complete expiration. This could generate auto-PEEP. However, in this study the end-expiratory pressure in the lung model was measured directly and the maximal difference from the set PEEP was < 0.3 mbar, which we consider not relevant.

Since the experiments with lung-healthy subjects were intended as a feasibility and utility study, we did not record spirometry data from the participants prior to the experiment. Potential participants with known lung disease were not included in the study cohort, and therefore we did not expect spirometry data to have any influence on the effect of the flow regulator. In addition, measurements were performed while breathing at rest, and no special breathing maneuvers such as forced inspiration or expiration were performed. However, for subsequent studies with patients suffering from pulmonary diseases, baseline spirometry data would be helpful for a correct interpretation of the results.

Regarding the fact that the volunteers reported mostly moderate discomfort of breathing with the flow regulator and discomfort was never intolerable we have to note that the observational period was short. However, we speculate that adaptation to the flow regulation may also enhance the perceived breathing comfort. However, a study with longer observational periods is needed.

Given the exploratory character of this study, future studies are needed to answer specific research questions. Concerning spontaneous breathing, an application of the device as a learning tool for the correct expiratory flow regulation in patients with obstructive lung diseases in comparison with other expiratory resistive devices seems favorable. Due to the increased EELV with flow regulation, an investigation of the preoxygenation period before the induction of anesthesia might reveal a benefit for patients, for instance a prolonged tolerance of apnea. Especially patient groups with risk factors for rapid deoxygenation may benefit form expiratory flow regulation. Concerning the intraoperative period, expiratory flow regulation might exert potential benefits in patients with obstructive lung disease and patients with risk factors for lung tissue atelectasis. For these patients, an intermittent post-operative application of flow regulation in comparison to non-invasive ventilation might reveal potential benefits.

## Conclusions

The here described flow regulator represents an easy to use and adjustable device for application of a self-regulated expiratory resistive load. Future applications of this flow regulation in spontaneously breathing patients and patients with mandatory ventilation alike may reveal potential benefits.

## Supplementary Information

**Additional file 1.** A-priori investigation of the mechanical characteristics of the flow regulator. **Figure S1**. Schematic drawing of the setup for the measurements with constant flow. **Figure S2**. A Flow trigger for decrease of the suspension angle α and B artificial airway resistance (R_AW_) for increasing aperture distances with a set flow of 600 ml·s^1^.

## Data Availability

The datasets used and/or analysed during the current study are available from the corresponding author on reasonable request.

## Abbreviations

EELV: End-expiratory lung volume
EEP: End-expiratory pressure
EIT: Electrical impedance tomography
FLEX: Flow-controlled expiration
MV: Minute volume
PEEP: Positive end-expiratory pressure
PLB: Pursed-lips breathing
P_lung_: Lung pressure
RR: Respiratory rate
T_Ex_: Expiratory time
T_In_: Inspiratory time
V_T_: Tidal volume
