# Supplementary material for: Control of the expiratory flow in a lung model and in healthy volunteers with an adjustable flow regulator: a combined bench and randomized crossover study
Source: Respir Res. 2021 Nov 14;22:292. doi: 10.1186/s12931-021-01886-7 (PMC8590868; doi:10.1186/s12931-021-01886-7)
Supplement: Supplementary file 1 — Additional file 1. A-priori investigation of the mechanical characteristics of the flow regulator. Figure S1. Schematic drawing of the setup for the measurements with constant flow. Figure S2. A Flow trigger for decrease of the suspension angle α and B artificial airway resistance (RAW) for increasing aperture distances with a set flow of 600 ml·s1. [file 12931_2021_1886_MOESM1_ESM.docx]

**Control of the expiratory flow in a lung model and in healthy volunteers with an adjustable flow regulator – a combined bench and randomized crossover study**

Johannes Schmidt^1^, Anna Martin^1^, Christin Wenzel^1^, Jonas Weber^1^, Steffen Wirth^2^ and Stefan Schumann^1^

^1^Department of Anesthesiology and Critical Care, Medical Center - University of Freiburg, Faculty of Medicine, University of Freiburg, Freiburg, Germany

^2^Department of Anesthesia, Intensive Care and Pain Medicine, Medical Center of the German Accident Insurance Institution, Murnau, Germany

Additional File 1:

The flow regulator’s characteristics were evaluated in a setting of constant flow (Figure 1). For determination of the flow trigger, a constant flow was set between 30 - 830 ml∙s^-1^ in steps of 50 ml∙s^-1^. Starting from the initial position, the angle of the plate was reduced down to α - 28.8° in 8 steps of 3.6°. For each angle setting, the flow trigger was defined as the flow which triggered a plate deflection. For determination of the flow resistance in dependence of the remaining aperture, a constant flow of 600 ml∙s^-1^ was set, sufficient to deflect the plate. Then, the respective screw was adjusted to reduce the aperture at the stopping point from 3.15 mm to 0.875 mm in 14 steps. For each setting, the device’s resistance was calculated. Each measurement was repeated three times.

Flow was determined utilizing a pneumotachograph (Type Fleisch 1, Dr. Fenyves und Gut, Hechingen, Germany). Airway pressures were measured before and after the flow regulator. The flow and pressure signals were recorded with a rate of 200 Hz by dedicated software (Labview Version 7.01, Austin, Texas, USA). All calculations were done offline utilizing MATLAB (Version 2018a, The MathWorks Inc., Natick, MA, USA).

The triggering flow could be adjusted between 633 ± 22 and 293 ± 14 ml∙s^-1^ and resistance could be adjusted from 1.0 ± 0.02 up to 40 ± 5 cmH_2_O∙l^-1^∙s^-1^ with a set flow of 600 ml∙s^-1^ (Figure 2).


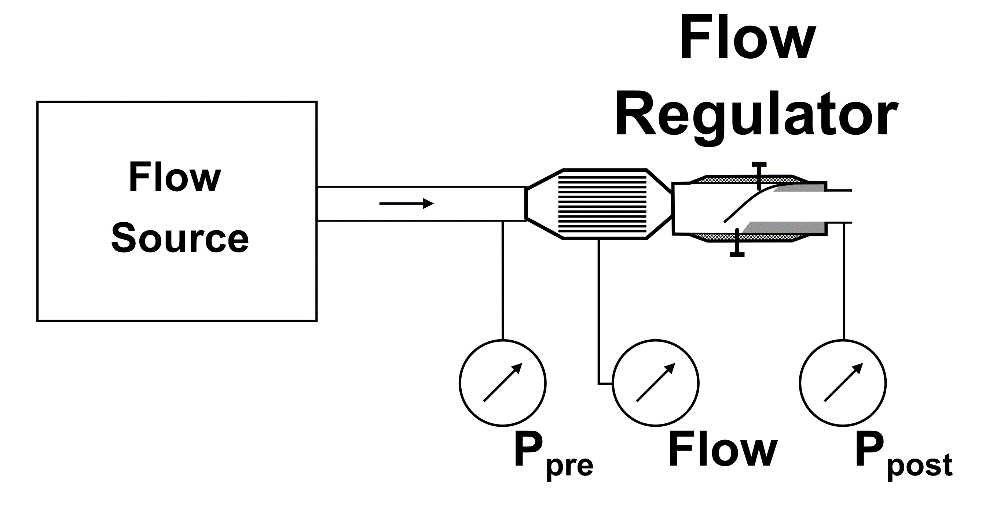


**Figure 1:** Schematic drawing of the setup for the measurements with constant flow. P_pre_: pressure in front of the flow regulator; P_post_: pressure behind the flow regulator.


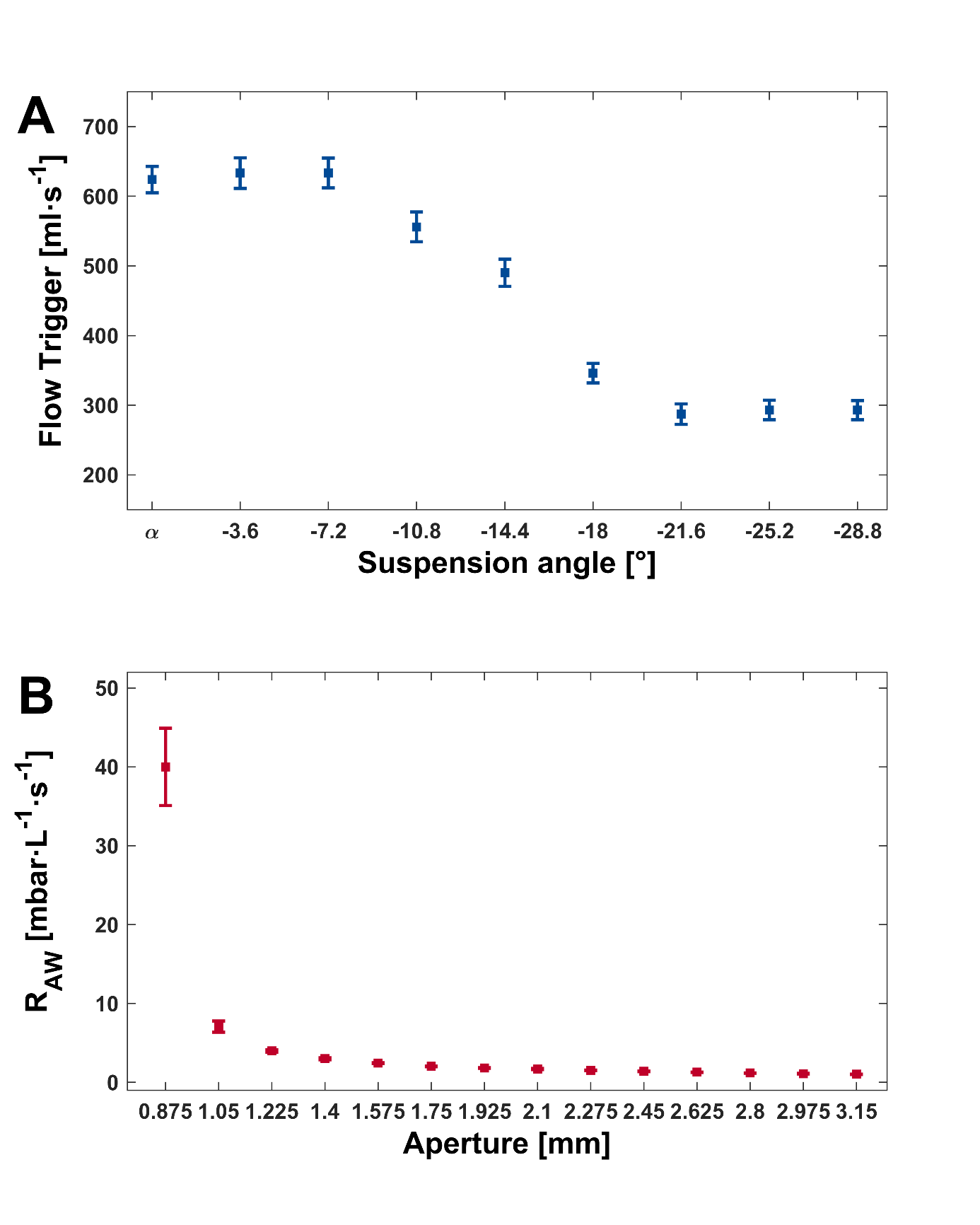


**Figure 2:** (A) Flow trigger for decrease of the suspension angle α and (B) artificial airway resistance (R_AW_) for increasing aperture distances with a set flow of 600 ml∙s^‑1^.
